# Is It Dengue?

**Published:** 1885-11

**Authors:** 


### Medical ★ Journal,

PUBLISHED MONTHLY AT

AUSTIN, TEXAS.

F. E. DANIEL, M. D., EDITOR AND PUBLISHER

Subscription Price: Two Dollars per Annum in Advance.

Papers for publication in this Journal should be in the hands of the Editor by the 10th of the month preceding the month in which it is wished to appear; they must be original,—never having appeared in print elsewhere, and must be contributed exclusively to this Journal.

Contributions to the department of Original Articles are cordially invited from the professiom of Texas, expecially. What is most desired is Clinical reports of cases, essays and short practical articles on any medical topic ; also solicited reports of proceedings of societies, clubs, etc., and items of medical news of general interest to the profession, such as notices of appointments of physicians, removals, changes, marriages, deaths, etc.

### EDITORIAL.

### IS IT DENGUE?

The epidemic which recently swept over Texas, sparing but few towns, was of so much graver type, and although in the main presenting the symptoms of dengue, yet was followed by sequellæ, or attended by complications so unusual, and so grave, as to excite in the minds of not a few, a doubt as to the identity of the disease. Indeed it seems to have been dengue *plus* something else, or dengue intensified and rendered more malignant by a poison which tended to decompose the blood, if dengue at all.

In a respectable per cent. of cases the clinical history of the disease as collated in the experience of the majority of the Austin physicians present at a meeting of Travis County Medical Society on the 5th, and that of other physicians in other parts of the state, is more like that of yellow fever than dengue. In one city in northeast Texas, where the disease seems to have been unusually severe, and even quite fatal, an old practitioner writes us that he has grave doubts as to the disease, and that in his experience it corresponds more closely to Pepper's description of relapsing fever than to dengue. This is a point, however, which could easily have been settled by the microscope, as *spirillæ* are said to be uniformly found in the blood in that fever. With reference to the tendency to disorganization of the blood, we quote the following from the Doctor's letter :

"The blood seems to me to be altered in its characteristics ; its coagulability seems to be wanting ; it appears to have lost its fibrin. The blood in hemorrages I have seen, was always fluid, and did not coagulate, after having been voided twenty-four hours. Its odor was peculiar. The sensation imparted to the touch when pressure was made upon an artery, I can only liken to the flowing of fluid through a gutta percha tube ; no decided impulse, but a gurgling sensation. Frequently, when the hemorrhages were controlled, there would be vomiting like that of black vomit in yellow fever. To my mind the matter thus throw up or passed from the bowels, was altered blood, mixed with mucus. It was exceedingly acid."

Black vomit has been observed in dengue, but it is so rare that Bemis says he never saw a case; but the unusual symptoms, sequellæ, etc., we had reference to, in addition to the vomiting of this altered blood, and as were described by the physicians present at the meeting referred to (and most of which are given in the paper of Dr. Burt which was read on that occasion and published in this number of the Journal),—were—a tendency to hemorrhage from any of the mucous surfaces ; a tendency to abortion in the pregnant state, and profuse hemorrhage following ; premature menstruation in young girls, and a hastening of the menses before the monthly crisis in woman, and the production of menorrhagia ; the formation of deep seated abscesses; parotitis, and (one case of ) synovitis, and suppuration of the joint ; kidney troubles, albumen and casts in the urine ; diarrhœa and pain in the abdomen ; delirium, and convulsions ; high temperature, 103 to 105, and in some cases, hyperpyrexia; extreme nervous prostration and general debility ; a great tendency to relapse, and at best a slow and painful convalescence.

Such complications or sequellæ did not occur in all cases, nor, indeed, were they characteristic of the epidemic; and the death rate, though there were some twelve deaths out of (estimated) twenty thousand cases in Austin, was even less than during other seasons ; yet the occurrence of such complications being so unusual, prompts us to the belief that some cause beside dengue was at work.

With regard to the eruption, it seems not to have been uniform either in its character or its time of appearance, occurring before, after, or during the fever, or not at all ; in some, papular, others, herpetic, miliary, or like urticaria ; but mostly like measles, the resemblance extending to the throat and eyes, and was such as to induce one to suppose it to be measles and not dengue.

Dr. McKinley, whose patrons are mostly colored, gives an interesting account of his practice. He had observed the disease in colored persons pretty much as it occurs in the whites, tendency to abortion and all ; but, with regard to the eruption, he says that whereas other physicians had 90 per cent. of the eruption, ninety per cent. of his cases did not have it ; that when observed, it was in those of mixed blood, and the admixture of white blood and the occurrence of the rash existing in direct ratio; while pure blacks had no eruption whatever.

An attack of the fever in the parturient state was attended with great danger, and we know of two women who died from the effects of the disease shortly after giving birth. In one, peritonitis set in, and she threw up a perfect specimen of the coffee ground black vomit,—clearly—altered blood. Under the microscope some days after, it was found to contain the bacteria of putrefaction, an abundance of the sugar fungi which resembled the ordinary yeast plant, and in which were found *vacuoles*.

To what influence are these rapid changes in the blood which cause its disintegration, and the death of the patient, due ? In yellow fever it is attributed to the presence of an organism which attacks the red cupuscle : why not in dengue ? In this connection the announcement that one of our Austin physicians has isolated a *micrococcus* and cultivated it, assumes vast importance; we await developments. This tendency certainly constitutes a grave source of danger not heretofore encountered or considered, and any measure or treatment looking to the maintainance of the integrity of the blood, has not, so far as we know, entered into the therapeutics of the fever. In the text books on physiology it is laid down that the integrity of the red corpuscle is maintained by the presence in the blood, normally, of .37 per cent. of *chloride of sodium*, but for which the corpuscles would be dissolved in the albumen of the blood. Had such solution taken place in the case of the man at the hospital described in Dr. Burt's paper? It would be interesting to enquire if this proportion of *sodium chloride* is diminished during the fever, and if so, it would seem to afford an indication for the administration of the chlorides or chlorine as a prophylactic or preventive of such solution and consequent disorganization of the blood. In yellow fever, chlorine in some form, enters largely into the treatment; but whether with a view to maintaining the normal standard of sodium chloride for the purpose of oxidizing the blood, we do not know, as the foregoing theory or hypothesis, is one of our own, and one which has never been mentioned in the books, so far as we are aware.

Another question of practical importance is,—to what are due the grave sequellæ and complications other than those blood changes, and which render the fever so grave, and even fatal in some cases? If it be dengue and is intensified, as we have intimated, by any atmospheric condition, especially if that atmospheric condition is due to any error of sanitation, it certainly behooves the medical profession to enquire into the cause, and advise steps for the removal of the source, to the end that we may escape another visitation, which may be even more malignant. In the absence of any positive knowledge on that head, and in view of the fact that the *turning up of soil* in hot weather is generally regarded as detrimental to health;—and in view of the *probability* that in the present instance it was a factor in the production of a condition of the atmosphere favorable to the spread of a disease of this character—the germ having been introduced—(as kindling to the match) it would be the part of ordinary common sense to interdict a repetition of it. Furthermore, in the event of the introduction of dengue here another year, the first case should be promptly isolated, and strict non-intercourse observed, as is the rule with other and known infectious diseases.

It would be a sad commentary on the intelligence—the civilization ot our people—were they to fail to profit by their recent fearful experience. Should another case of this fever *be brought here*, as was the first case this season, surely our people will not be so indiscreet as to institute and keep up a social and friendly intercourse with the patient, and thus spread the infection;—each new case becoming a focus, from which radiation of the poison occurs, till the whole atmosphere is charged with it, and an epidemic lighted up. Another epidemic, even though it be dengue, which it seems is not the harmless ailment it was formerly supposed to be, would do much to retard the growth and development of our fair city and grand Empire of the West. Verily, "experience *is* a dear teacher"—but, they say—some people will learn in no other school.

